# Expanding Access to MRI: The Role of All-Purpose Mid-Field and 1.5-T Scanners

**DOI:** 10.1148/radiol.251406

**Published:** 2025-09-02

**Authors:** Vikas Gulani, Devasenathipathy Kandasamy, Andrew G. Webb, Derek K. Jones, Raju Sharma

**Affiliations:** ^1^Department of Radiology, Michigan Institute for Imaging Technology and Translation, University of Michigan, 1500 E Medical Center Dr, B1G503, Ann Arbor, MI 48103; ^2^Department of Radiodiagnosis and Interventional Radiology, All India Institute of Medical Science, New Delhi, India; ^3^Department of Radiology, Leiden University Medical Center, Leiden University, Leiden, the Netherlands; ^4^Cardiff University Brain Research Imaging Centre (CUBRIC), School of Psychology, Cardiff University, Cardiff, United Kingdom

## Introduction

The year 2023 marked the 50th anniversary of the publication of the landmark paper by Lauterbur, describing zeugmatography, now known as magnetic resonance imaging, or MRI (1). From the start, the community of MRI scientists has shown immense creativity, pushing the field in numerous scientific and clinical directions that have had great impact, so much so that in 2001, cross-sectional CT and MR imaging were called the most important medical innovations in 50 years, in a survey of physicians (2). Imaging technology now touches almost all facets of medicine. Transformative progress has been made in magnet design, image acquisition, image reconstruction, and scientific and clinical applications; the technology has been continuously improved and reinvented.

However, the high cost of MR equipment has strongly influenced who has access. The cost of MRI technology remains very high, creating a barrier to entry both to working scientifically in the field and to accessing the technology for a vast majority of the world. Scanner density per 1 million inhabitants in various countries, according to the Organisation for Economic Cooperation and Development, ranges from 57.39 to 0.24 (3). The parts of the world located in resource-constrained environments, dominated by the global South, fall at the lower end of this scale and lack access to MR imaging (3). This remains a challenge for the MR community which has prided itself on innovation but has not yet addressed this problem, which also extends to many rural and low-income urban communities in high-income countries (HICs) (4).

## Low-Field Scanners

Recently, there has been a strong movement toward reimagining MRI hardware costs, with an eye toward increased access by broadening the usable magnetic field spectrum. Teams have created permanent magnet scanners dedicated to head-only use at low fields and low cost, some with extreme portability, and clinical needs entirely defined locally. One system operating at low field has even opened the capability of whole-body scanning, though at lower spatial resolutions than current clinical standards (5). Low field is defined here arbitrarily as lower than 0.1 T, reflecting a number of emerging systems operating in this field range. Two scientists working on creating accessible MR technology (6) have highlighted five challenges for MR researchers to address: *(a)* improve low-field technologies, *(b)* build physicians’ confidence *(c)*, prioritize open-source approaches, *(d)* develop artificial intelligence tools for low- and middle-income countries (LMICs) contexts, and *(e)* boost funding for sustainable MR imaging (7). These are excellent points and should be followed earnestly. Perhaps the biggest advantage of this mindset is that it allows investigators to develop previously unexplored designs and use cases for MRI, effectively designing directly for resource-constrained environments and addressing unique needs. An example is hydrocephalus in local pediatric populations, which is difficult to follow clinically without advanced imaging and can easily be followed with low-field machines and little need for high resolution or high signal-to-noise ratio.

A criticism leveled at these machines (in this case, in the African context), is that “they are merely a Band-Aid, a temporary fix in a dynamic economic environment; a technology that undervalues Africa’s potential” (8). Stated differently, the technology being offered is critical and important, but still fundamentally different to that accessed in higher-resource settings, in that it cannot be used for multiple body part scanning at current clinical standard spatial resolutions, including routine brain, spine, body, and musculoskeletal imaging. Providing very low-cost machines with significantly fewer clinical roles does provide access that may support research endeavors or enable diagnosis of diffuse disease not requiring high spatial resolution, but it does not do so in a manner that promotes a second goal of health equity among populations. Given current technology and that available for the foreseeable future, there will be a significant struggle in achieving sufficient spatiotemporal resolution with this approach alone to detect small focal pathologic features (especially small tumors, infarcts, etc); conversely, it may become more readily possible to use these technologies as gateways to determining if advanced imaging is needed (9).

## Mid-Field Scanners

An additional direction of development is in what can be termed the “mid-field” realm, defined here as 0.1–1.0 T, which is re-emerging as a viable range for general-use machines. Traditional manufacturers such as Siemens Healthineers, GE HealthCare, and Philips Healthcare have demonstrated use of mid-field, 0.55-, 0.5-, and 0.6-T magnets, respectively (10–14). The goals of these systems include expanding access to MRI technology to populations currently unable to afford it. While the GE HealthCare and Philips Healthcare systems are not commercially available, there are over 200 installations of the Siemens 0.55-T system family in Latin American, Association of Southeast Asian Nations, and African settings. The platform fulfills the criterion of a clinical all-purpose machine with lower siting requirements, a lower total cost of ownership than 1.5-T systems, and a broad application portfolio. Hardware costs are not transparently published, but for context, we estimate cost of purchase of commercially available systems operating at low field (<0.1 T) to be approximately $80 000–$600 000, mid-field to range from $300 000 to $700 000, and 1.5 T to be greater than $1 million. Cost of ownership is complex and varies by location, though at least the cost of cryogens has been essentially eliminated due to the introduction of low-helium-volume units with no boiloff, at the expense of increased power consumption.

Safety considerations for both mid-field and higher-field magnets are similar, with projectile risk being a significant concern. Tragic accidents (15) highlight the need for vigilance regarding MRI safety both in HIC and LMIC settings, but MR safety–related infrastructure and education may not be readily available in the latter and thus presents another consideration in the placement of scanners.

Significant initial clinical experience with commercially available mid-field systems has been published (10,11,16–21). Studies in the brain, spine, knee, and abdomen have shown capabilities to make key diagnoses are preserved in mid-field magnets with gradient and receiver arrays designed to minimize cost, though there are certainly opportunities to improve current image quality. Diagnostic capability is the critical issue facing clinical radiologists, whether in LMIC or HIC settings.

Members of our own team have worked to install and operationalize a 0.55-T system in a low-income rural Indian setting, without access to advanced imaging. The project was approached as a prototype for expansion of access using mid-field and lower-cost high-field general-use scanners in LMICs around the world. In 2020, the department of radiodiagnosis at the All India Institute of Medical Sciences (AIIMS) in New Delhi partnered with Siemens Healthineers and the University of Michigan to install a 0.55-T, 60-cm bore magnet designed for the rural site at the Comprehensive Rural Health Services Project (CRHSP; Ballabgarh, an outpatient and emergency medical center associated with AIIMS and located 2–3 hours’ drive outside of Delhi). The CRHSP otherwise lacks cross-sectional imaging such as CT and provides near-free care to the regional community. The goals of the project include identifying and solving the cultural, regulatory, technical, and clinical workflow barriers to providing such access.

The successful launch of the program required government assistance for land and power facilities for the scanner, appropriate siting permissions, and legal releases for the project which were accomplished through collaboration with the departmental and institutional leadership at AIIMS New Delhi, in collaboration with the CRHSP. Further cultural concerns that the local community would feel exploited with a short-term benefit were allayed with the help of CRHSP, in the form of a minimum 3-year placement of the magnet. Due to space and access constraints, a relocatable container unit was used to site and operate the magnet, equipment, and operator rooms in one combined, self-contained unit, easing construction and infrastructure constraints. Due to regular power outages experienced in the area, uptime of the system was ensured by including a diesel generator and uninterrupted power supply units in the setup to cover for short-term power outages (<2 hours). For longer power outages, the system is capable of automatic ramp-down (approximately 30 minutes) that protects the magnet from damage, and to reramp the magnetic field (approximately 4 hours) after power returns.

Two radiographers designated to operate the system had no prior MRI scanning experience and 1 week of training, with artificial intelligence–based scan assistance on the machine designed to decrease training requirements and ease the scanning process with a varied case mix (22). Protocoling, monitoring, and interpretation were provided remotely by the AIIMS New Delhi site. Scans were provided free of cost to patients who were referred by the outpatient center of the CRHSP. Initial experience in 413 patients showed that approximately 10% of patients responding (36 of 379) would have either not gotten or may not have gotten their study if not for the availability of the scanner onsite. The remainder would have gotten the scan elsewhere if recommended by their physician, but the cost (₹5000–₹7000 [U.S. $60–$85] for a noncontrast scan) would be substantial for the patient population in this region, where a 2022 survey of 4000 households reported a monthly average family income of approximately ₹17 000 (U.S. $200). Despite the lower field and lack of a trained radiographer, image quality was rated by interpreting radiologists as “good” in 98% (407 of 413) patients, and “average” in the remaining 2%. It should be stressed that while the population in this region does at least have access to other scanners at higher cost, many other areas or countries suffer from a total lack of access. Systems such as this, enabling general-use scanning, could be partnered with low-field portable systems to expand access to many populations currently lacking these services. The clinical interplay between the still-developing portable low-field technology and general-use 0.5-T to 1.5-T technology remains to be determined due to the still-evolving use cases of portable low-field scanning.

### 1.5-T Scanners

Multiple companies building 1.5-T scanners have arisen in China, the pricing of which is not clear. In India, new vendors such as Voxelgrids are attempting to produce 1.5-T whole-body magnets. Project SAMEER, sponsored by the government of India, is attempting to create an Indian-designed, general-use magnet with all components designed in India and a sustainable industry and infrastructure around it (23,24). These movements could have high impact, though neither Voxelgrids nor SAMEER are available commercially.

An important question that arises for LMICs is whether refurbished or older 1.5-T scanners could be used instead of newer scanners. However, there is a well-described problem that when equipment is past its serviceable life and sent to LMICs, out-of-date and unusable equipment can become a burden for the recipient countries to dispose of, resulting in “equipment graveyards” that are antithetical to the goals of the movement (25). Thus, new equipment, or equipment with significantly extended serviceable life (and available service) is needed.

We note that field strengths higher than 1.5 T are less relevant in the accessibility context, though they are important for some diagnoses and especially research. 3-T systems for focused clinical applications, and higher-field systems for research, could be available in major cities, where such facilities are best utilized.

## Recommendations and Conclusion

Based on initial experience, just as for recommendations for expansion of access for very-low-field scanners (7), the authors would like to offer key recommendations for the expansion of 0.5-T to 1.5-T scanners in underserved populations:

1. Scanners compliant with local clinical regulations should be clinically and commercially available for routine patient scanning.
2. Governmental cooperation should be sought for site selection and availability and for power access for any general-use scanner, and to help overcome regulatory hurdles for scanning.
3. The cooperation of local clinical and government leadership should be sought to identify and address cultural concerns of patient populations.
4. Scanners should be capable of performing the most common clinical examinations (ie, head, spine, musculoskeletal, body).
5. While some manufacturers are taking commendable steps in reconfiguring workflows and supporting local infrastructure, others emphasize accessibility only in rhetoric. Companies can develop profitable business models for LMICs and simultaneously be true partners in global radiology. To do this, their business strategies must evolve to address local needs and include pricing flexibility, service models tailored to resource-constrained contexts, and collaborative innovation ecosystems.
6. Scans should be simple to perform by less-trained technologists and require minimal support.
7. Scanners should be resilient to deal with potentially challenging environmental conditions (ie, with respect to electrical supply and cryogen requirements) and have pathways to service for longer-term maintenance.
8. Major radiologic and MRI organizations should play a leadership role by making archived MR safety training material freely available to resource-constrained settings, host regular accessible webinars on MR safety protocols and incident prevention specifically geared toward LMIC teams, as well as engage these communities via continuous dialogue and knowledge exchange on developing best safety practices.
9. Radiologists should be available for protocoling and image interpretation.

Our recommendations are broadly aligned with those made for the development and dissemination of low-field systems (7). However, while these scanners could create vital new capabilities and use cases (especially in screening for diffuse disease), allow placement in locations too remote for maintenance or operation of superconducting magnets, and help triage patient populations away from or towards advanced imaging, they will not soon provide the range of diagnostic capabilities that make general-use mid- and high-field MRI so versatile and clinically important.

If MR is to become both accessible and contribute to equitable health outcomes, the full range of field strength as a variable must be explored with general-use 0.5-T to 1.5-T magnets vital to providing equitable access.
